# Preclinical assessment of potential interactions between botulinum toxin and neuromodulation for bladder micturition reflex

**DOI:** 10.1186/s12894-015-0048-z

**Published:** 2015-06-09

**Authors:** Xin Su, Angela Nickles, Dwight E. Nelson

**Affiliations:** Medtronic plc, Neuromodulation Research, 7000 Central Avenue, Minneapolis, MN 55432 USA; Physiological Research Laboratories, 7000 Central Avenue, Minneapolis, MN 55432 USA

**Keywords:** Electrical stimulation, Micturition, Spinal nerve, Botulinum toxin

## Abstract

**Background:**

While botulinum toxin A (BoNT-A) has become a more commonly used second-line treatment for patients with detrusor overactivity, it remains unknown whether the impacts of this therapy may persist to influence other therapies such as sacral neuromodulation. In this preclinical study we have evaluated urodynamic functions to intradetrusor injection of BoNT-A and the bladder inhibitory effects of spinal nerve stimulation (SNS) following BoNT-A treatment.

**Methods:**

Female rats were anesthetized with 3 % isoflurane. BoNT-A (2 units, 0.2 ml) or saline were injected into the detrusor. Rats then were housed for 2 days to 1 month before neuromodulation study. Monopolar electrodes were placed under each of the L6 spinal nerve bilaterally under urethane anesthesia. A bladder cannula was inserted via the urethra for saline infusion and intravesical pressure recording.

**Results:**

Intradetrusor injection of BoNT-A for 1–2 weeks or 1 month significantly increased bladder capacity compared with saline injection (*p* < 0.05, two-way ANOVA). Following BoNT-A, SNS attenuated the frequency of bladder contractions, either eliminating bladder contractions or reducing the contraction frequency during electrical stimulation. Inhibition of the contraction frequency by SNS following BoNT-A treated rats was not different from that measured following saline injection.

**Conclusions:**

BoNT-A increased the bladder capacity, but compensating for additional saline infusion to the enlarged urinary bladder in BoNT-A pretreated rats, the bladder contractions induced by bladder filling were attenuated by SNS. BoNT-A did not alter the ability of SNS to inhibit bladder contraction following intradetrusor injection of BoNT-A for 2 days, 1–2 weeks or 1 month. These results support further pre-clinical and clinical studies to evaluate potential interactions or combination therapy with neuromodulation and intradetrusor BoNT-A therapeutic approaches.

## Background

Detrusor overactivity is a common medical problem resulting in increased voiding frequency, urinary urgency and urge incontinence. Anti-muscarinic drugs are generally used as first line therapies while mirabegron is a first-in-class β3-adrenoceptor agonist licensed and has shown to be effective and have a good safety profile [1]. If medications cannot be tolerated or do not provide sufficient symptomatic relief, the most common second line treatment options are 1) sacral neuromodulation (InterStim® Therapy) via stimulation of the spinal nerve (S3), which is efficacious in 70-80 % population of patients with urge incontinence and increased frequency [2]; and 2) intradetrusor injection of botulinum toxin type A (BoNT-A) which improves detrusor overactivity and increases bladder capacity [3, 4].

Urodynamic studies have shown BoNT-A injection to increase maximum cystometric capacity and bladder compliance [5–10]. With the increasing use of BoNT-A, many patients may receive both of these second line therapies. Retrospective study has shown that patients who are dissatisfied with or fail BoNT-A treatment with a mean interval of 23 months between the BoNT-A and the sacral neuromodulation test stimulation, respond successfully to InterStim Therapy [11]. Systematic studies of potential interactions of sacral neuromodulation and BoNT-A have not been performed. One unknown question relates to the potential impact of prior BoNT-A treatment upon subsequent neuromodulation. Specifically, does prior treatment with BoNT-A alter patient success rates to neuromodulation therapy? No clinical or preclinical data is available to address this question from either efficacy or safety perspectives.

We have utilized the rat model of the bladder micturition reflex contraction (BRC) to screen for potential interactions between BoNT-A and neuromodulation [12–14]. Spinal nerve stimulation (SNS) results in an inhibition of the frequency of BRC. Stimulation parameters (e.g. stimulation frequency and current intensity) have been optimized systemically. Our previous work in the rat [15, 16] demonstrates a positive intensity-dependent bladder inhibitory effect in response to electrical stimulation of the L6 spinal nerve. In the present study, we characterized the relationship between current intensity of SNS and bladder contraction frequency in rats pretreated with intradetrusor injection of either saline or BoNT-A.

## Methods

### Animals and study design

A total of 79 female Sprague–Dawley rats, 200–300 g were used, 44 in the BoNT-A treatment group (intradetrusor injection) and 35 in the saline treatment group. After 2 days to 1 month of the treatment, all rats were anesthetized with urethane and examined urodynamically to determine the bladder compliance or bladder micturition reflex following the treatment. The inhibitory effects of SNS on BRC were also studied. During the urodynamic study, rats were maintained at 37 °C with a heating pad (Viking Medical, Medford Lakes, NJ) and were euthanized by CO_2_ asphyxia upon completion of experimental procedures. The experimental protocols were approved by the Medtronic Institutional Animal Care and Use Committee as well as the Non-clinical Research Board (Minneapolis, MN).

### Intradetrusor injection

Rats were anesthetized with 3 % isoflurane. The urinary bladder was exposed via a small suprapubic incision under sterile conditions. Two units (0.2 ml) of BoNT-A (BOTOX®, manufactured by Allergan, Besse Medical, West Chester, OH) or saline were injected into the whole detrusor randomly and evenly (10–16 μl per site) through a 30-gauge needle. The same volume of normal saline was injected into the detrusor at the same positions as a control group. The rectus fascia was closed with an absorbable suture, and the skin was closed with silk sutures. Animals then were housed for 2 days (9 in saline and 10 in BoNT-A), 1 week (11 in saline and 15 in BoNT-A) or 2 weeks (4 in saline and 6 in BoNT-A, grouped together with 1 week treatment), or 1 month (11 in saline and 13 in BoNT-A) before the urodynamic studies.

### Neuromodulation of L6 SNS

Rats were anesthetized with urethane (two i.p. injections, 4 min apart, total 1.2 g/kg, 200 mg/ml in saline, Sigma-Aldrich, St. Louis, MO). To deliver electrical stimulation, the skin around the dorsal sacral and thoracic area was shaved and a dorsal midline incision was made from approximately L3 to S2, the L6/S1 posterior processes were exposed. The S1 processes were removed and the L6 nerve trunks were localized caudal and medial to the sacroiliac junction. Electrodes were made from teflon-coated, 40-gauge, stainless steel wire with the coating removed from a 0.5 cm section (Cooner Wire Co., Chatsworth, CA) and was placed under each of the L6 spinal nerve bilaterally. After the wire electrode(s) were positioned, silicone adhesive (Kwik-Cast, World Precision Instruments, Inc, Fl, USA) was applied to cover the wire around the nerve, and the skin incision was sutured shut. The electrode(s) were connected to a Grass S88 stimulator (Grass Medical Instruments), through stimulus isolation unit(s) (SIU-BI, Grass Medical Instruments). A needle electrode under the skin of the tail served as the ground.

To record bladder contractions, a polyethylene cannula (PE50) was inserted into the bladder via the urethra, and secured with a suture tie. The urethral cannula was connected via a T- connector to a pressure transducer (MLT0380D, ADInstruments, Colorado Springs, CO, USA) and data acquisition system (ML880/P, ADInstruments). The intravesical pressure signal was amplified for recording (ML228, ADInstruments). The other end of the T-connector was attached to a syringe pump.

### Urodynamic studies

To induce BRC, saline was infused into the bladder via the syringe pump at a rate of 50 μl per minute to induce a micturition reflex (here defined as a bladder contraction of a magnitude >10 mmHg). The infusion rate was then lowered to 10 μl per minute and continued until 3–5 consecutive contractions were established. At this time, the saline infusion was terminated, and the BRC continued.

Electrical stimulation of the spinal nerve evoked hind-toe twitches and/or pelvic floor muscle contraction. In each rat, the motor threshold current (T_mot_) was defined as the lowest current required to evoke the first, barely observable, muscle contraction. Biphasic pulses (pulse width 0.1 ms) of different intensities (0.8 x T_mot_, T_mot_ and 2 x T_mot_) were applied at a frequency of 10 Hz to stimulate the spinal nerve.

After a 15 min control period, nerve stimulation was applied; the BRC were recorded for additional 20 min post stimulation. Initially, single current intensity stimulation was applied for a total of 10 min (20 in saline and 25 in BoNT-A) in one rat. In some other experiments (15 in saline and 19 in BoNT-A), all intensities in the order 0.8 x T_mot_, T_mot_ and 2 x T_mot_ for a total of 15 min were tested in the same rat. Since there is no difference in sensitivity to nerve stimulation between two stimulation modalities [14–16], the data were grouped according the test intensity.

### Data analysis

To evaluate the bladder compliance/capacity, a few cystometry parameters were assessed once the first micturition reflex was established including basal bladder pressure (BP, mmHg), threshold pressure (TP, mmHg, pressure before the micturition occurred), and bladder capacity (ml, volume infused to induce a micturition reflex, interval x 0.05 μl•min-1). The bladder compliance was assessed mathematically using the capacity divided by the difference of BP with TP. Once the stable BRC is established, two parameters of BRC were evaluated: frequency/interval and amplitude between the maximum pressure reached during voiding and after voiding in mmHg in 5 min bins. When the BRC was completely suppressed by high intensity stimulation, the frequency of the bladder contraction was designated “0” and amplitude data for that interval was excluded. All data were compared to the mean response during the last 5 min prior to stimulation.

All data are expressed as mean ± SEM. Results were analyzed with two-way ANOVA with Bonferroni post-hoc test by Prism 5 (GraphPad Software, Inc., San Diego, CA). A value of *p* < 0.05 was considered statistically significant.

## Results

**Fig. 1:**
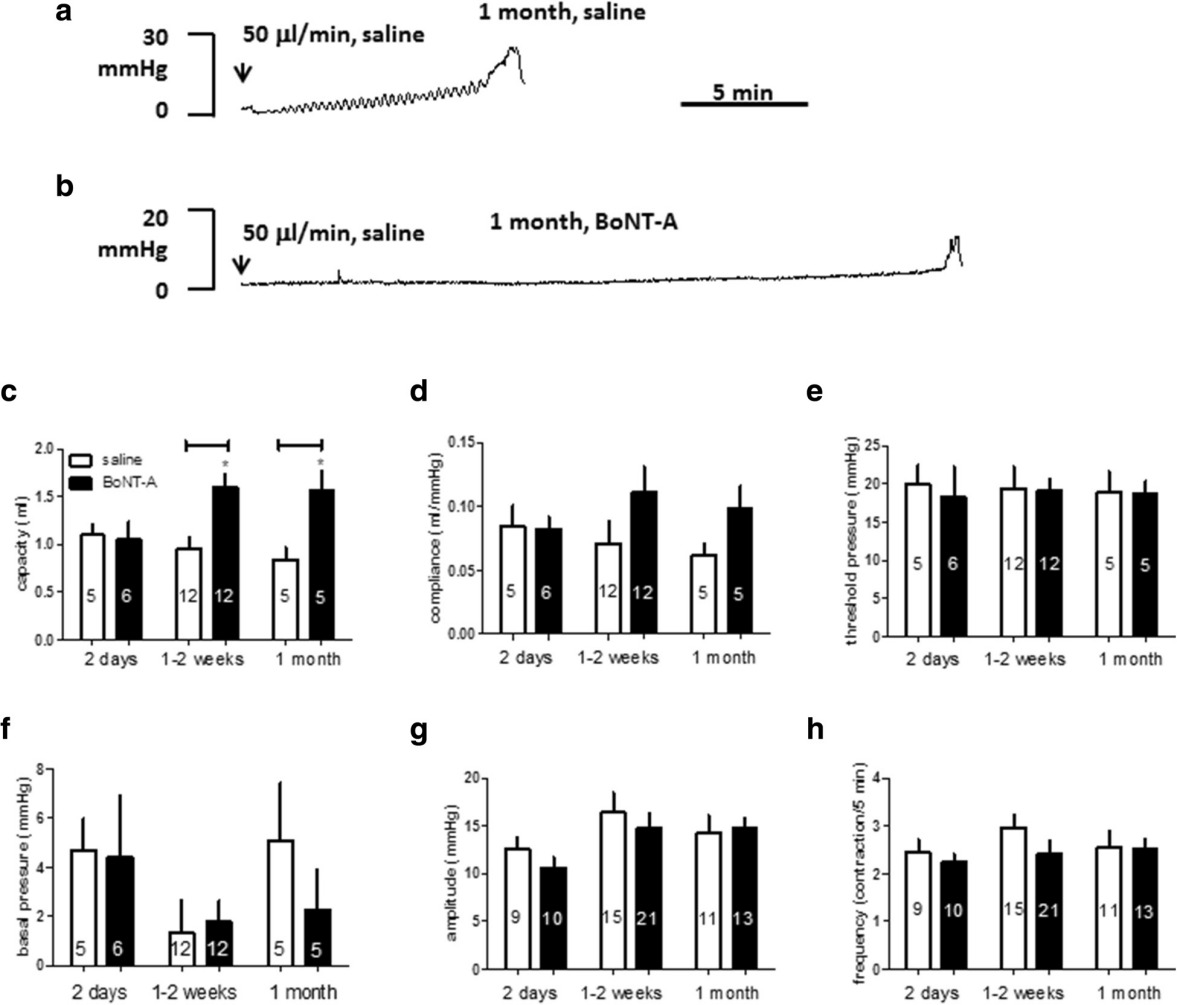
Urodynamic function in rats pretreated with saline or botolinium toxin A (BoNT-A). **a** and **b**. Raw traces of intravesical pressure recording to intravesical saline infusion, 1 month following with saline or BoNT-A injections. **c**-**f**. Summary of cystemetry parameters before the first micturition contraction occurred following saline infusion in rats with intradetrusor injection of saline and BoNT-A. **g**-**h**. Basal amplitude or frequency of bladder contractions once bladder micturition reflex contraction was established in saline or BoNT-A pretreated rats. *p < 0.05, Two-way ANOVA, Bonferroni post test. The number of animals is indicated in each symbol

Following treatment of BoNT-A or saline, infusion of saline into the bladder at a constant rate induced an increase in bladder pressure. Until the TP for urination is reached, only a small increase in pressure was observed. Figure 1a and b shows the two traces of intravesical pressure from rats 1 month post intradetrusor injection of either saline or BoNT-A. More saline infusion was required to evoke the bladder micturition reflex in the rat pretreated with BoNT-A. Summarized data in Fig. 1c demonstrate that intradetrusor injection of BoNT-A for 1–2 weeks or 1 month significantly increased bladder capacity (*p* = 0.008, two-way ANOVA). We found no differences on compliance, TP and BP between the control and BoNT-A groups (Fig. 1d, e and f). Continuing the saline infusion (see Methods) resulted in BRC. Once the BRC was established with large volume infusion in BoNT-A treated rats, the baseline values of contraction amplitude and contraction frequency from BoNT-A treated rats were not different from that from saline treated rats (Fig. 1g and h).

**Fig. 2:**
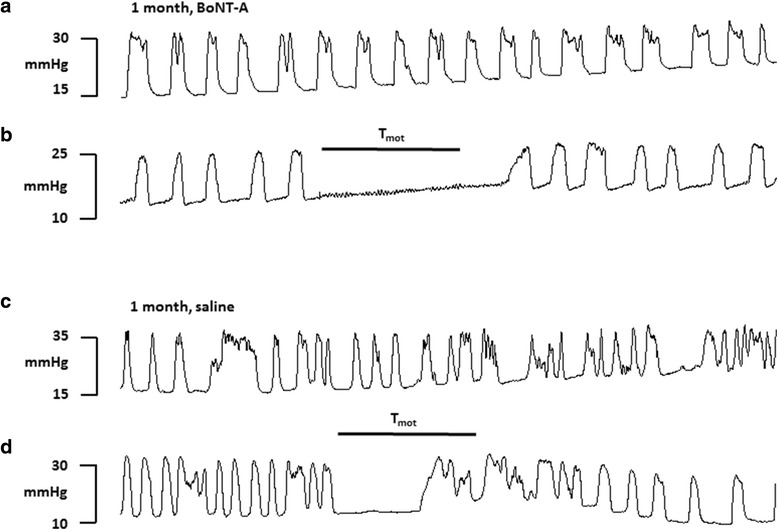
Typical experimental records showing the bladder micturition reflex contraction (mmHg) 1 month following either BoNT-A (**a** and **b**) or saline (**c** and **d**) intradetrusor injection without stimulation (**a** and **c**) or to L6 spinal nerve stimulation at motor threshold (T_mot_) intensity (10 Hz, pulse width 0.1 ms) for 10 min (**b** and **d**). Horizontal bars indicate duration of spinal nerve stimulation

Without stimulation the BRC was similar following saline or BoNT-A pretreatment (Fig. 2a and c). When electrical stimulation of the spinal nerve was delivered it attenuated the frequency of bladder contractions, either eliminating bladder contractions completely or reducing the contraction frequency during electrical stimulation in both BoNT-A and saline treatments (Fig. 2b and d). The contraction frequency returned to the prestimulation value after termination of electrical stimulus.

**Fig. 3:**
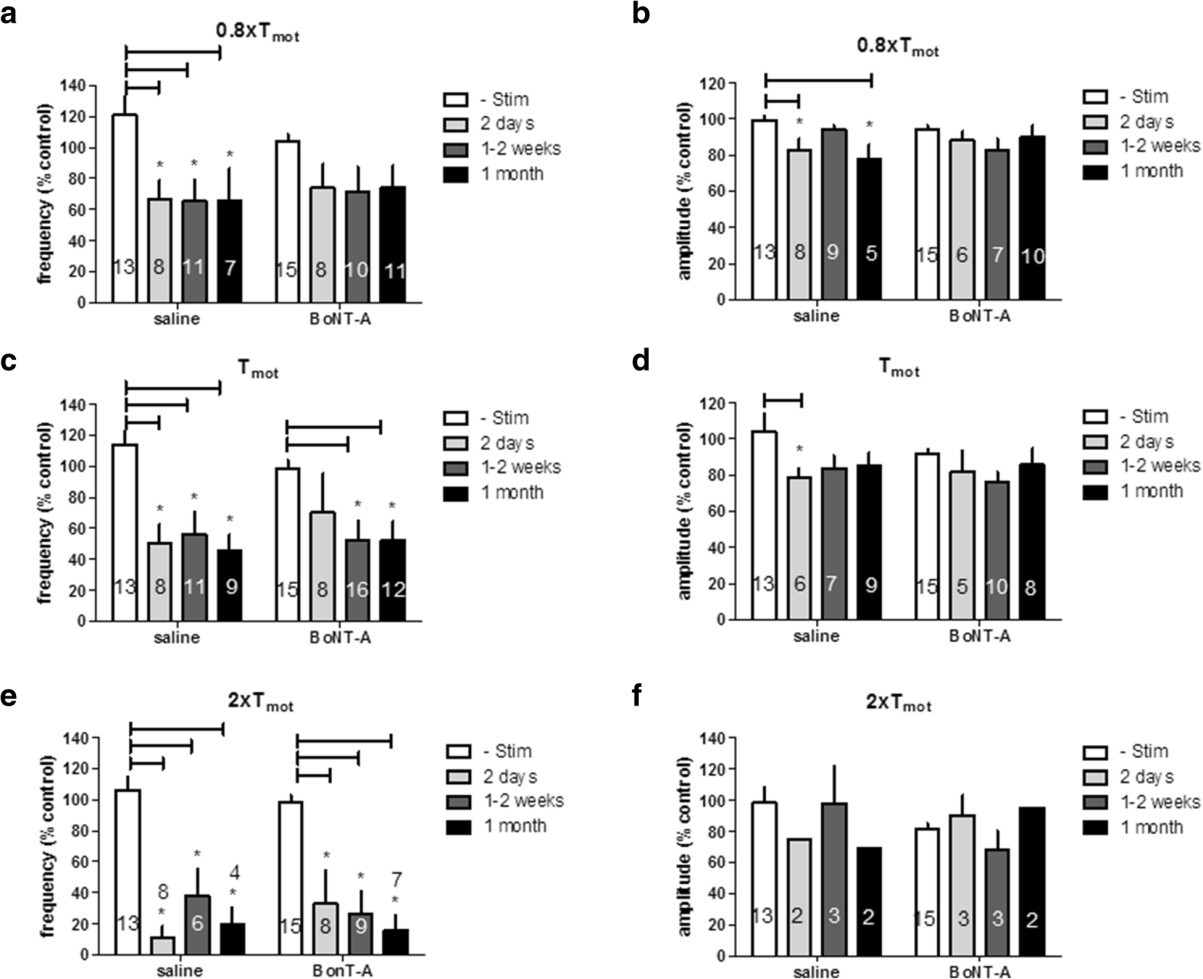
Effects of spinal nerve stimulation on the frequency (**a**, **c** and **e**) and amplitude (**b**, **d** and **f**) of the bladder micturition reflex contraction at intensities relative to the motor threshold (T_mot_) intensity (10 Hz, pulse width 0.1 ms). Responses are represented as a percentage of control (%control), where the baseline response before stimulation is defined as 100 %. * *p* < 0.05, Two-way ANOVA, Bonferroni post test. The number of animals is indicated in each symbol

Figure 3 summarizes the mean responses of BRC responses during different intensities of SNS in saline or BoNT-A treated rats. Two-way ANOVA analysis demonstrates that BRC frequency was significantly decreased by SNS at all current intensities tested following either BoNT-A or saline injections (*p* < 0.05, vs time control when stimulation was not applied).

Two days, 1–2 weeks and 1 month post BoNT-A injection, SNS at T_mot_,10 Hz decreased the frequency of contractions to 70.18 ± 25 % (n = 8), 52.14 ± 12 % (n = 16, *p* < 0.05), and 51.94 ± 12 % of controls (n = 12, *p* < 0.05, vs. 98.77 ± 5 % control without stimulation), respectively. Using the same stimulation parameters, SNS decreased the frequency of contractions in saline pretreated rats, to 50.32 ± 12 % (n = 8, *p* < 0.05), 55.93 ± 15 % (n = 11, *p* < 0.05), and 45.45 ± 10 % of controls (n = 9, *p* < 0.05, vs. 114.16 ± 8 % control without stimulation), respectively. Inhibition of the contraction frequency in BoNT-A treated rats was not different from that measured in saline treated rats (*p* > 0.05).

SNS elicited relatively weak and inconsistent inhibition on the amplitude of the bladder contractions (*p* < 0.05, vs time control when stimulation was not applied, two-way ANOVA). Figure 3b, d and f summarize effects of SNS on amplitude of BRC.

## Discussion

We have previously reported that SNS inhibits the frequency of volume induced BRC, and that 10 Hz is the optimally efficacious stimulation frequency in this model system [15]. The inhibitory effects of SNS on bladder contractions have also been shown to be intensity-dependent, and the magnitude of the inhibition increases with the applied current (stimulus intensity, 16). In the present study, we used 10 Hz stimulation frequency and studied the intensity-response function of the bladder micturition reflex to SNS in BoNT-A or saline treated rats to establish the sensitivity of neuromodulation in bladder control. Our data do not show a difference in the magnitude of the inhibitory effect of neuromodulation between BoNT-A and saline treated rats.

Intradetrusor injection of the BoNT-A dose used in our study (2 units) was shown in a prior study to not cause urinary retention in the rat [8]. Consistent with these results, we did not observe any reduction in the amplitude of bladder contraction following BoNT-A treatment. The interval between BoNT-A injection and urodynamic evaluation ranged from 2 days to 1 month. BoNT-A significantly increased bladder capacity, 1–2 weeks and 1 month following injection. This is consistent with the time-course for the onset and duration of the effect of BoNT-A by others. Significant inhibitory effects on neurotransmitter release and urodynamic function were observed 5–30 days after injection [7, 17]. Clinically, patients experience relief of sudden urges to urinate, and a reduction in urine leakage starting several days to 2 weeks following BoNT-A injection. In patients the effect has been show to persist for 3 to 9 months [18].

In our tests the effectiveness of BoNT-A alone was demonstrated by its action on increasing bladder capacity. This is consistent with studies reported by others regarding effects of BoNT-A alone in the rat with different manipulations [6, 8, 9]. In spinal cord injured rats, BoNT-A attenuated non-voiding contractions [9]. In addition, BoNT-A has been also reported to suppress acetic acid induced bladder hyperactivity and inflammatory reactions [6, 8]. We did not examine the immunohistochemistry and histology changes on the detrusor following BoNT-A injection in the present study. Others have reported that the mechanism of BoNT-A on bladder control may be related to its inhibition on neurotransmitter release [17, 19–22]. This is reflected by an increase in bladder capacity. Clinically BoNT-A results in an increase in cystometric capacity. In studies reported increases of 60 % ([23], spinal cord injured patients with detrusor overactivity), 35-80 % ([24], pediatric neurogenic bladder), and 100-200 % ([25], neurogenic detrusor overactivity patients) have been observed.

Intradetrusor injection of BoNT-A increases bladder capacity. Additional saline infusion was able to compensate the enlarged urinary bladder in BoNT-A pretreated rats and establish the BRC. Although the bladder capacity is increased, the frequency of bladder contraction in our model is not decreased, and the ability of neuromodulation to inhibit BRC is unaffected. In the present study, SNS produced an equal degree of bladder inhibition following either saline or BoNT-A treatment. The magnitude of inhibition was comparable to that reported previously in this model [15, 16]. Therefore, in BoNT-A treated rats, SNS produced a bladder inhibition that occurred independent of the increased bladder capacity produced by BoNT-A. It is likely that BoNT-A acts via interference with the release of neurotransmitters from peripheral nerve terminals and the response of afferent activated dorsal horn neurons [26, 27] while neuromodulation produced an inhibition of the BRC via an action at a higher level CNS on the neuronal control of the bladder reflex (e.g. the pontine micturition center and cerebral cortex, [28]). If BoNT-A and SNS have different sites of action, it would be logical that these therapies may have independent effects on bladder inhibition.

The present results are not definitive. Detrusor overactivity is often a multi-etiological disease. Our data were obtained in normal and anesthetized rats. Only BRC, not cystometric capacity was measured. However, inhibition of the BRC is a consequence of an increased bladder capacity [29]. Since pretreatment with BoNT-A does not appear to attenuate the bladder inhibitory effects of neuromodulation, it is reasonable that an equally increased bladder capacity would be observed in rats pretreated with intradetrusor injection of either BoNT-A or saline. In support, from a clinical observation patients who are dissatisfied with or fail BoNT-A treatment respond successfully to InterStim Therapy [11]. The neuromodulation was evaluated following a single injection of BoNT-A in this work. Our findings encourage further pre-clinical studies evaluating the combination of neuromodulation and repeated intradetrusor injections of BoNT-A on urodynamics in animals with normal and irritated bladder as well as controlled clinical evaluations of therapy interactions or combinations using sacral neuromodulation with BoNT-A in patients with detrusor overactivity.

## Conclusions

Our study confirmed previous observations that intradetrusor injection of BoNT-A increases bladder capacity in the rat. This increased capacity in BoNT-A treated rats does not influence the frequency of the BRC. Regardless of the interval between injection and stimulation, treatment with BoNT-A does not appear to influence the ability of SNS to inhibit BRC. In future studies it will be important to test whether neuromodulation is also efficacious in BoNT-A treated patients, and whether the two treatments could be combined in the treatment of patients with refractory detrusor overactivity.

## Abbreviation

BoNT-A: Botulinum toxin A
BRC: Bladder rhythmic contraction
SNS: Spinal nerve stimulation
T_mot_: Motor threshold
BP: Basal bladder pressure
TP: Threshold pressure
